# Dealing with the Surgical and Medical Challenges of Penetrating Brain Injuries

**DOI:** 10.1155/2013/209750

**Published:** 2013-01-13

**Authors:** Nikolaos Syrmos, Mario Ganau, Antonella De Carlo, Lara Prisco, Laura Ganau, Vasileios Valadakis, Kostantinos Grigoriou, Charalampos Iliadis, Dimitrios Arvanitakis

**Affiliations:** ^1^Neurosurgery Department, Venizeleio General Hospital, 71409 Heraklion, Greece; ^2^Neurosurgery Department, University of Verona, Piazzale Stefani 1, 37126 Verona, Italy; ^3^Intensive Care Department, University College Hospital, NHS Trust, London NW1 2BU, UK; ^4^School of Medicine, University of Cagliari, 09124 Cagliari, Italy

## Abstract

Peacetime has reduced the overall incidence of penetrating brain injuries (PBI), and those related to missile penetration are not common anymore at least in western countries. Nevertheless, PBI still occur, and car crashes or work accidents are their main causes. The management of such cases is characterized by many challenges, not only from a surgical and medical point of view, but also for the different and sometimes bizarre dynamics by which they present. Herein we report an unusual deep penetrating brain injury, due to a high-energy crash against a metallic rod in a construction site, with a good surgical outcome despite dramatic clinical conditions on admission. A discussion of the surgical results and functional outcome related to PBI, as found in the English medical literature, is provided. Moreover the most common postoperative complications along with the diagnostic flow charts and therapeutic options useful to prevent inappropriate treatment are highlighted.

## 1. Introduction

Peacetime has reduced the overall incidence of penetrating brain injury (PBI), and those related to missile penetration are not common anymore at least in western countries. Nevertheless, PBI still occur, often with a bizarre presentation and car crashes or work accidents being their main causes. 

 In 2009, Pascual et al. presented the case of a 32-year-old woman with a penetrating orbital-cranium injury caused by a metallic rod while working in her kitchen [1]. Gutiérrez-González et al. in 2008 had described a rare case of self-inflicted trauma caused by an electrical drill [2]. Selvanathan et al. in 2005 reported the case of a penetrating cerebral-cranium injury caused by a nail gun [3]. Herein we present another unusual case of severe head trauma caused by the deep penetration of a metallic rod through the frontal bone till to the temporal lobe. We discuss the surgical results and functional outcome related to unusual deep PBI as found in the English medical literature with the aim to identify the most common postoperative complications along with the best therapeutic options to prevent inappropriate treatment. 

## 2. Case Presentation

A 19-year-old motorbiker accidentally lost control of his bike, while riding without wearing his helmet, and crashed against the wall of a building in course of construction. In the accident he was projected against an iron rod protruding from the building, which deeply penetrated into his head. The patient's GCS score upon arrival of the ambulance was 4/15 (E1, V1, M2); due to the complex and bizarre traumatic modality, at their arrival the paramedics were obliged to remove the rod from the wall before stabilizing the patient who was thereafter sedated, intubated, and ventilated. He appeared substantially unstable from a hemodynamical perspective (arterial blood pressure of 100/70 mmHg; pulse rate 120 bpm; breathing rate 20 apm), but a blood sample for hemogas analysis resulted normal. 

**Figure 1:**
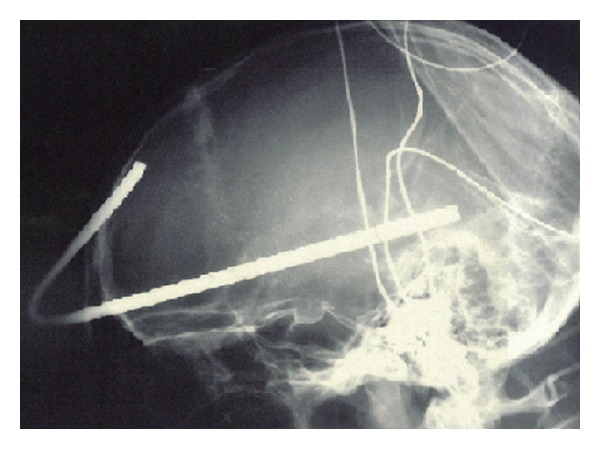
Lateral plain X-ray of the skull demonstrates the trajectory of the metallic rod, which enters approximately 1 cm above the supraorbital rim, involves the frontal sinus, and points through the right temporal lobe for an intracranial length of 14.5 cm.

**Figure 2:**
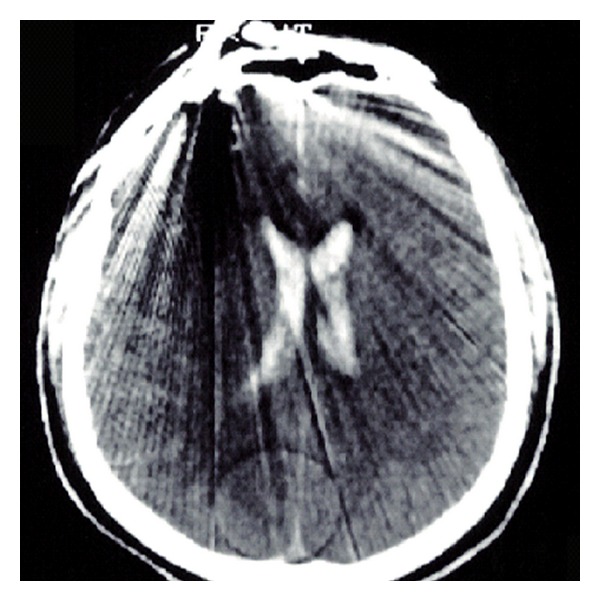
Preoperative axial CT scan of the head shows the entry point of the metallic body, causing radiographical artifacts. The frontal horn damage can be suspected because of the bilateral intraventricular hemorrhage.

**Figure 3:**
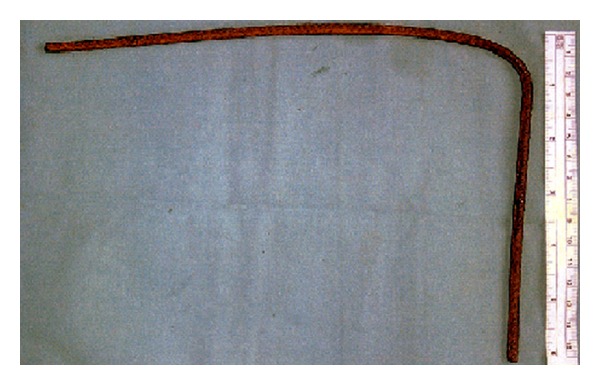
Postoperative photograph of the metallic rod, which had a diameter of 6 mm and a length of 35.5 cm.

**Figure 4:**
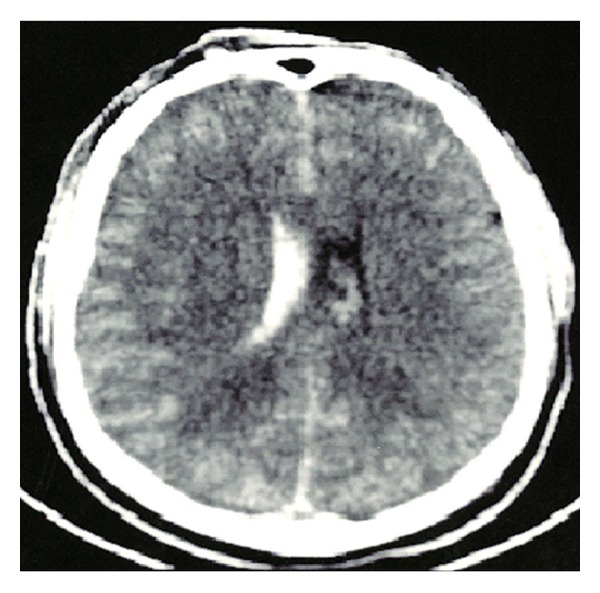
Postoperative axial CT scan demonstrates residual contusions in the right frontal lobe and in particular the presence of blood in the ventricular system.

He was immediately transported to the closest trauma center and underwent urgent angio-CT head scan which detected a severe blunt PBI in the forehead caused by a 6 mm (diameter) × 35.5 cm (total length) iron rod, entering approximately 1 cm above the supraorbital rim, involving the frontal sinus, and pointing through the right temporal lobe for an intracranial length of 14.5 cm (Figures 1 and 2). Hopefully, no cerebral vascular injuries or concomitant extracranial blunt traumas were highlighted. Urgent surgical removal and debridement was performed by enlargement of the entry point through a small right frontal craniectomy. The rod and the small bone fragments which were found within the damaged brain tissue were taken out easily (Figure 3). Gentle debridement of devitalized brain was performed using a combination of suction and irrigation. A small blood clot was also found and removed. Meticulous hemostasis was achieved using the bipolar diathermy and instillation of warm normal saline solution. Intraoperatively, the right frontal horn resulted perforated; therefore an external ventricular drainage with intracranial pressure (ICP) monitoring was inserted (first measurement: 22 mmHg). A watertight dural closure and obliteration of frontal sinus with a pericranium flap were realized in order to prevent cerebral infection and CSF fistulae. Perioperatively and postoperatively a broad-spectrum antibiotic therapy was administered, along with continuous infusion of Sodium Valproate. A postoperative CT head scan (Figure 4) demonstrated few right frontal lobe cerebral contusions without any mass effect; blood was also found within the ventricular system, the subarachnoid space, and the basal cisterns. 

The antibiotic and antiepileptic prophylaxis was continued during the rest of his stay in the ICU; two cerebrospinal fluid cultures resulted negative, and after the stabilization of the ICP the EVD was removed. In the sixth postoperative day the patient developed aspiration pneumonia which delayed the weaning process, and a tracheotomy was deemed necessary to protect his airways. Three weeks later, he was transferred to the ward with a GCS of 12/15 (E3, V4, M5) and no evidence of neurological focal signs. He was lately discharged home with a Glasgow Outcome Scale (GOS) of 4 (moderately disabled) due to mild cognitive impairment associated with concentration difficulties. Because of that he had undergone for the following 3 months an intensive rehabilitation training with psychological assistance, since then he has been followed up for more than a year and so far he has not developed any neurological complication or behavioral problem. From the descriptions of his relatives he now lives a normal life. 

## 3. Discussion

In western countries PBI are generally caused by metallic objects, or low velocity missiles [1, 4–7]. Four major modalities of PBI may be identified: urban violence, road traffic accidents, home accidents, and suicide attempts. Noteworthy, a significant relation between age, comorbidity, and outcome has been outlined, suggesting that mortality is higher in patients over 50 years of age. Whilst, many PBI are incompatible with life leading to death even before hospitalization, other patients with mild to moderate injuries may be saved if appropriate treatment protocols are adopted [8, 9]. In fact as in traumatic brain injuries, low GCS on admission and intractable raises in ICP are acutely detrimental; on the other hand hypotension together with coagulopathy and respiratory distress is the main mechanism of secondary insult engendered by PBI [10, 11]. Thus aggressive treatment of secondary mechanisms of injury must be initiated, and the patient must be monitored closely in order to avoid possible sudden cardiac or pulmonary complications [12]. 

Surgical intervention as soon as possible, in combination with aggressive intensive care management, is the goal in the management of such patients and already has significantly reduced the mortality and morbidity associated with these injuries. Nevertheless, from a review of the literature a considerable variability emerges among neurosurgeons attitude on the most appropriate treatments of PBI; in particular, wide variations exist in the amount of surgical debridement performed and the use of ICP monitoring. For instance, Martins et al. did not recommend surgical treatment in PBI patients with a GCS score of 3–5/15 in the absence of hematoma causing a mass effect [9]. However their recommendation is limited to gunshot wounds and cannot be applied to every case of PBI, since other cases, including the one herein reported, demonstrate that a good clinical outcome cannot be excluded despite deep penetration of foreign bodies or dramatic clinical conditions on admission [1–3]. As Gutiérrez-González et al. suggest, the permanent neurological deficit seems related to the degree and location of the primary injury; this and the absence of concomitant blunt traumas could explain the successful clinical outcome observed in our patient [2]. 

To date central nervous system infections are the commonest local complications, risk factors include deep or multifocal brain injury, intracranial retained bone and metallic fragments, CSF fistulae, and air sinus involvement [13, 14]. Accurate debridement and abundant intraoperative irrigation with saline solution are mandatory, since they represent the first step to effectively remove possible retained bacteria, fungi, or other pathologic agents. Obliteration of the frontal sinus with a pericranium flap is advocated as the treatment of choice to prevent CSF fistulae and postoperative infections; watertight dural closure may or may not be performed according to the type of trauma and surgical procedure (craniotomy or craniectomy) performed [15, 16]. Moreover, the use of local hemostats should be carefully tailored to reduce the risks of foreign body reaction or mass effect [17]. An issue raised by many authors is related to the use of antibiotic and antiepileptic prophylaxis which remains controversial, but also the use of hyperventilation, hypothermia, and steroids are still open to debate, since no Level A or B evidence is available regarding those arguments due to the lack of appropriately conducted randomized controlled trials [18]. 

To summarize, the management of PBI has evolved along the decades as we have gained significant experience and knowledge not only in military medicine but also in general neurotraumatology [14]. Whereas some extreme positions or pragmatical choices must be considered, as Wani et al. suggested that in developing countries with limited resources, PBI patients in comatose condition should not be managed aggressively if the brain damage is multifocal, and irreversible shock or hemodynamic instability is noticed [19], both, the experience so far acquired with such complex and often bizarre PBI as well as the medical and surgical therapeutic armamentarium available to date induce to concretely hope that the number of PBI patients effectively treated and saved across the globe will further grow in the near future.
